# CORRELATES OF ELEVATED C-REACTIVE PROTEIN AMONG BLACK OLDER ADULTS: EVIDENCE FROM THE HEALTH AND RETIREMENT STUDY

**DOI:** 10.1093/geroni/igac059.879

**Published:** 2022-12-20

**Authors:** Heather Farmer, Courtney Sinclair Tobin, Roland J Thorpe, Jr.

**Affiliations:** University of Delaware, Newark, Delaware, United States; Fielding School of Public Health, University of California Los Angeles, Los Angeles, California, United States; Johns Hopkins University, Baltimore, Maryland, United States

## Abstract

Substantial evidence documents gender and racial disparities in C-reactive protein (CRP), a measure of systemic inflammation, among older adults. Yet, the comparative approaches of these studies may obscure distinct risk and protective factors associated with elevated CRP among older Black Americans. To pinpoint opportunities for intervention, this study utilizes a “within-group approach” to identify the sociodemographic, psychosocial, behavioral, and health-related determinants of elevated CRP among older Black women and men. The sample consisted of 2,420 Black respondents aged 51+ in the Health and Retirement Study (2006-2016). Gender-stratified, random effects logistic regression models were used to examine determinants of elevated CRP (>3.0 mg/L). More than 50% of Black women had elevated CRP, and we found that younger age, Medicaid, lower mastery, religiosity, overweight/obesity, physical inactivity, and ADLs contributed to elevated CRP among this group. In contrast, elevated CRP was reported among only 37.25% of Black men, for whom financial distress was associated with lower odds of elevated CRP; religiosity, less neighborhood cohesion, current smoking, overweight/obesity, ADLs, and more chronic conditions were associated with greater odds of elevated CRP among this group. Sociodemographic factors had a limited association with elevated CRP among older Black Americans. Rather, a range of psychosocial, behavioral, and health-related factors were more influential determinants of elevated CRP among older Black Americans. Most notably, findings demonstrate distinct determinants of CRP among Black women and men, underscoring the critical need to further evaluate the risk and protective mechanisms undergirding disparities among this aging population.

